# Supplementary figures and images for: Live attenuated vaccination protects aged chimeric ACE2 mice from severe SARS-CoV-2 pathogenicity in vivo
Source: PLoS Pathog. 2026 Apr 22;22(4):e1014167. doi: 10.1371/journal.ppat.1014167 (PMC13132463; doi:10.1371/journal.ppat.1014167)

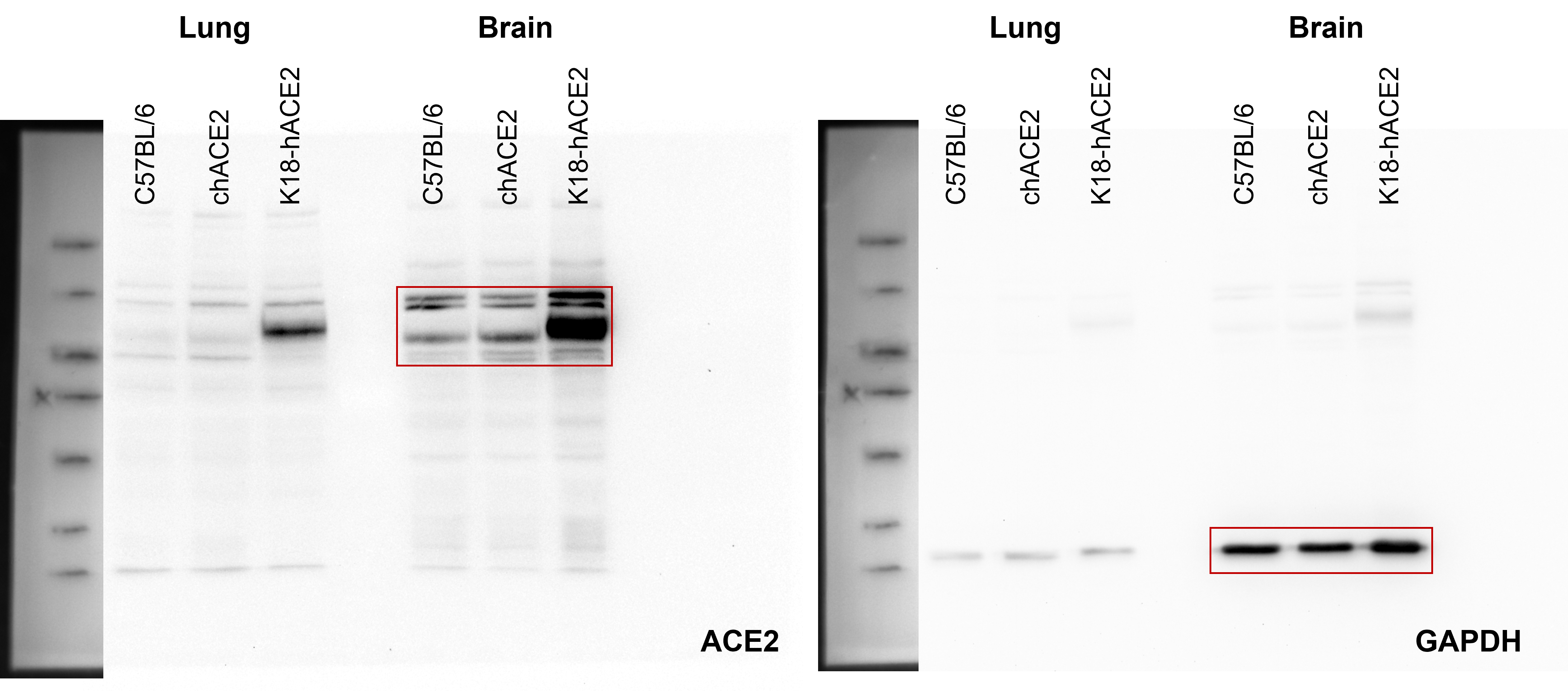

Supplement: S1 Raw Images — (ZIP) [file ppat.1014167.s002.zip › Fig.2D Brain.tif]

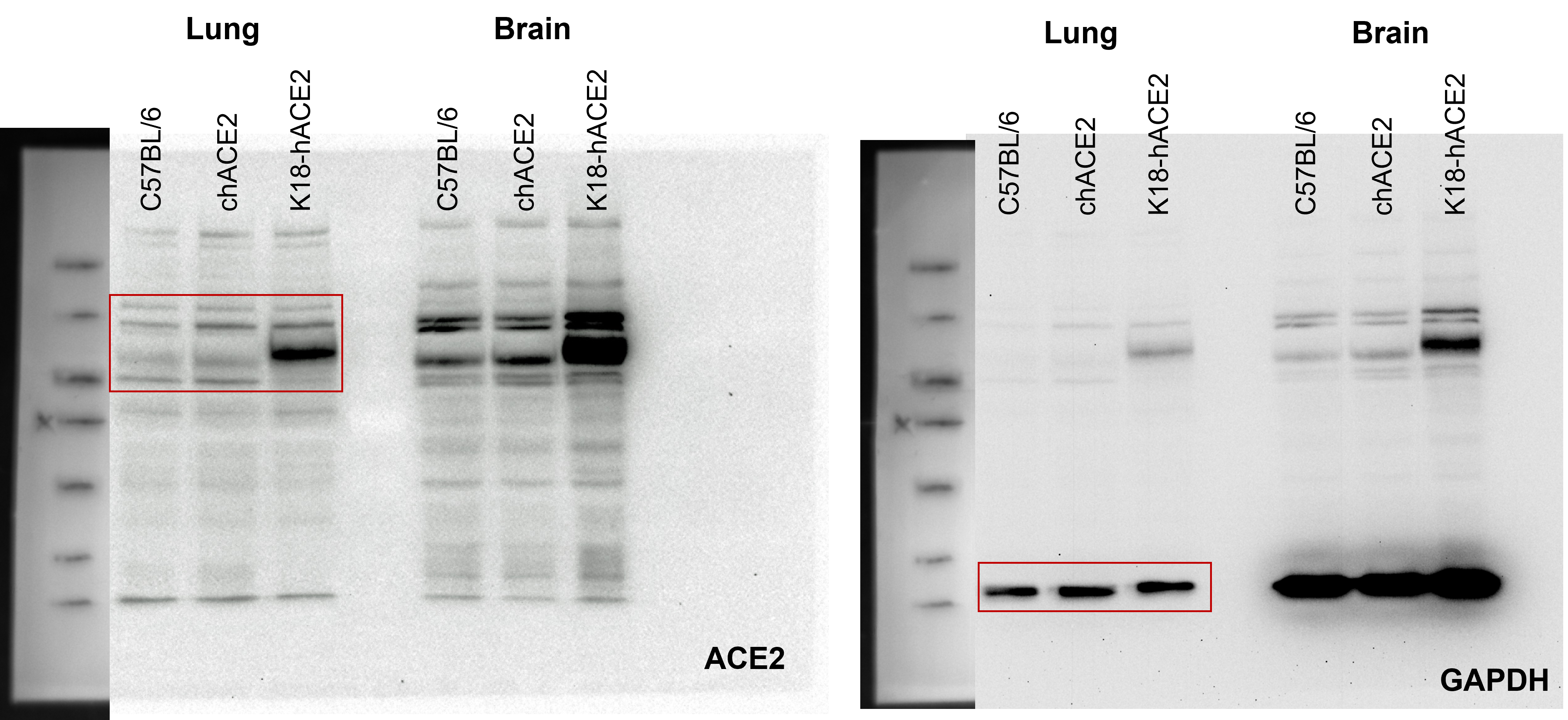

Supplement: S1 Raw Images — (ZIP) [file ppat.1014167.s002.zip › Fig.2D Lung.tif]

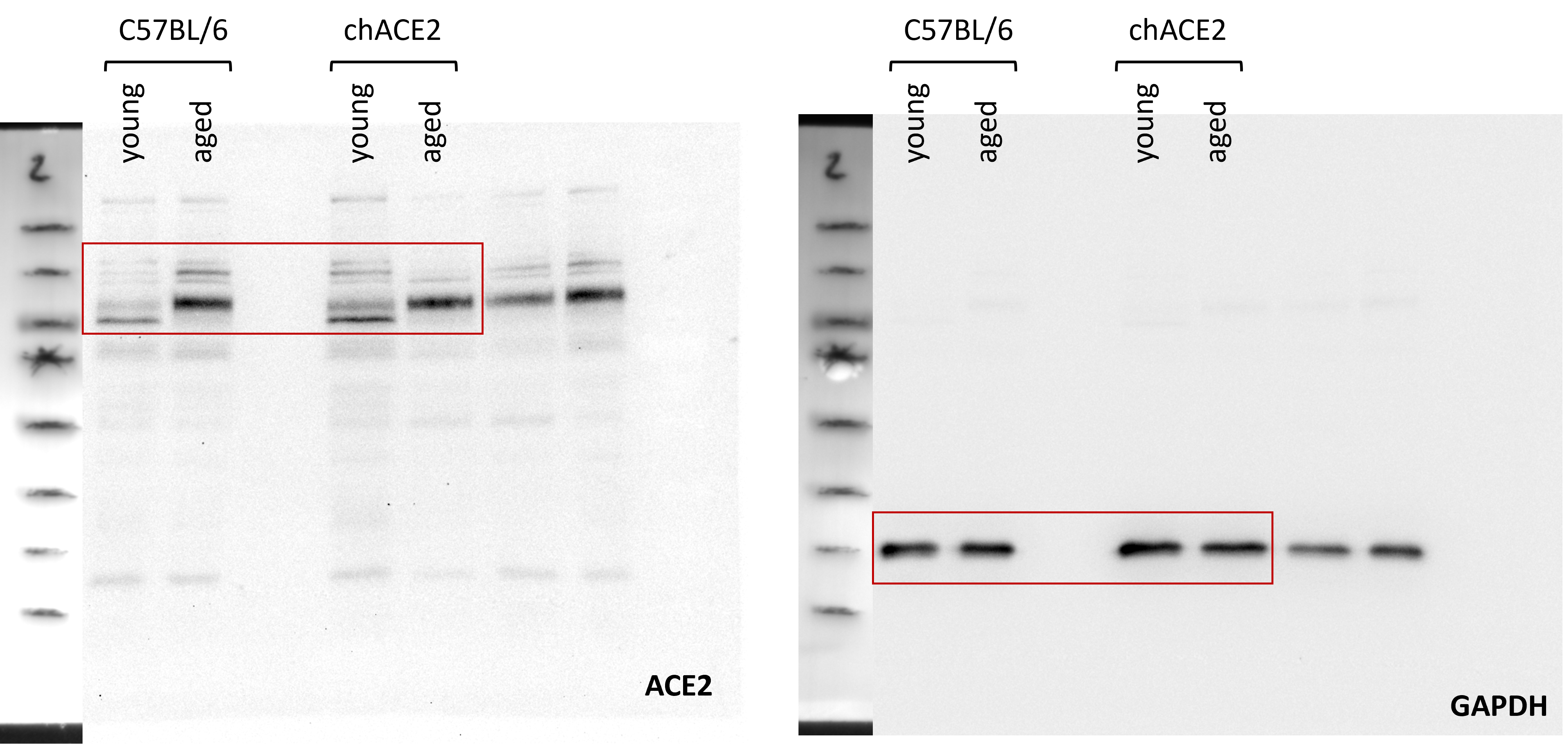

Supplement: S1 Raw Images — (ZIP) [file ppat.1014167.s002.zip › Fig.6H.tif]

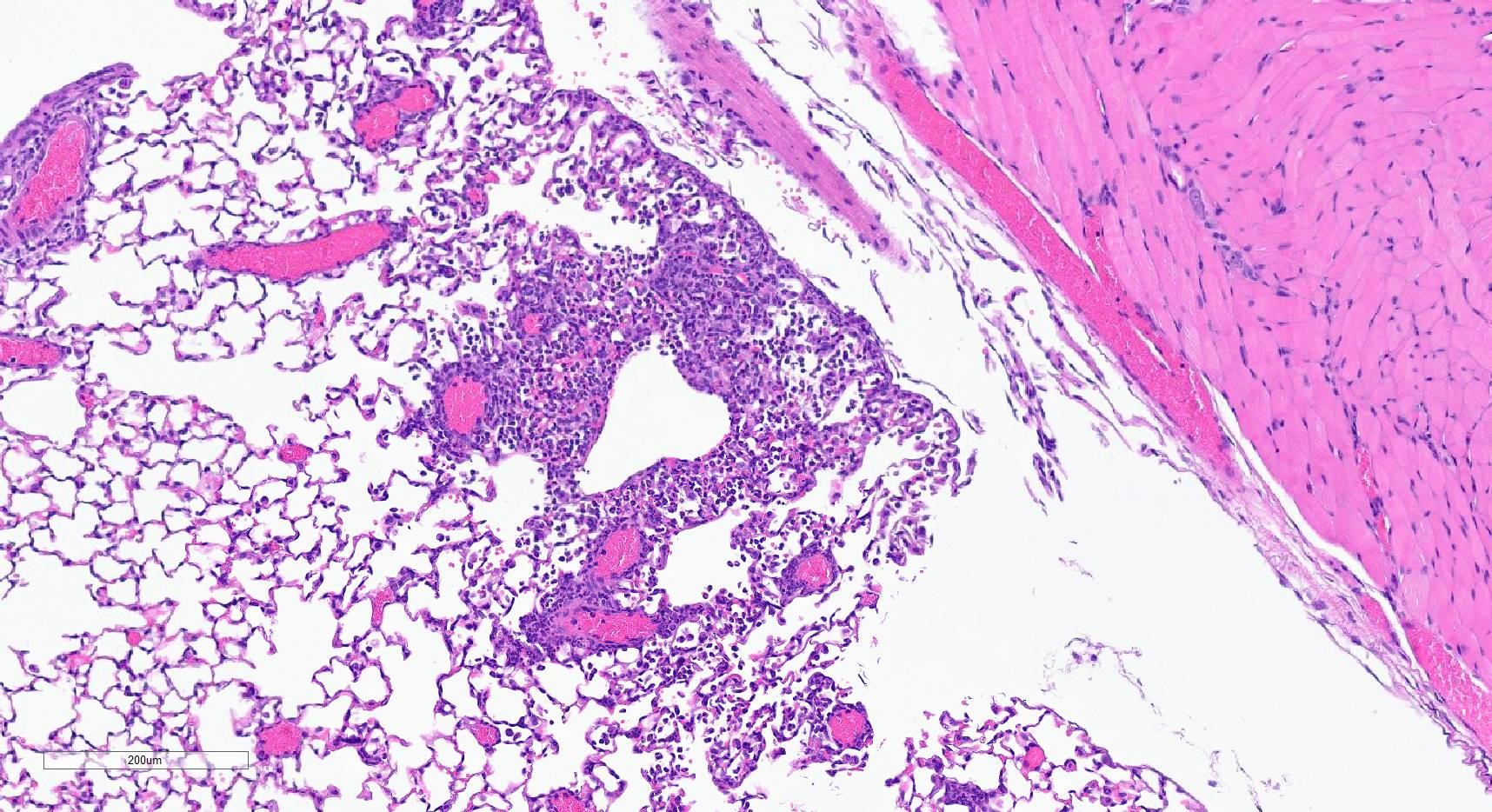

Supplement: S1 Raw Images — (ZIP) [file ppat.1014167.s002.zip › Fig.8A aged LAV zoom in.tif]

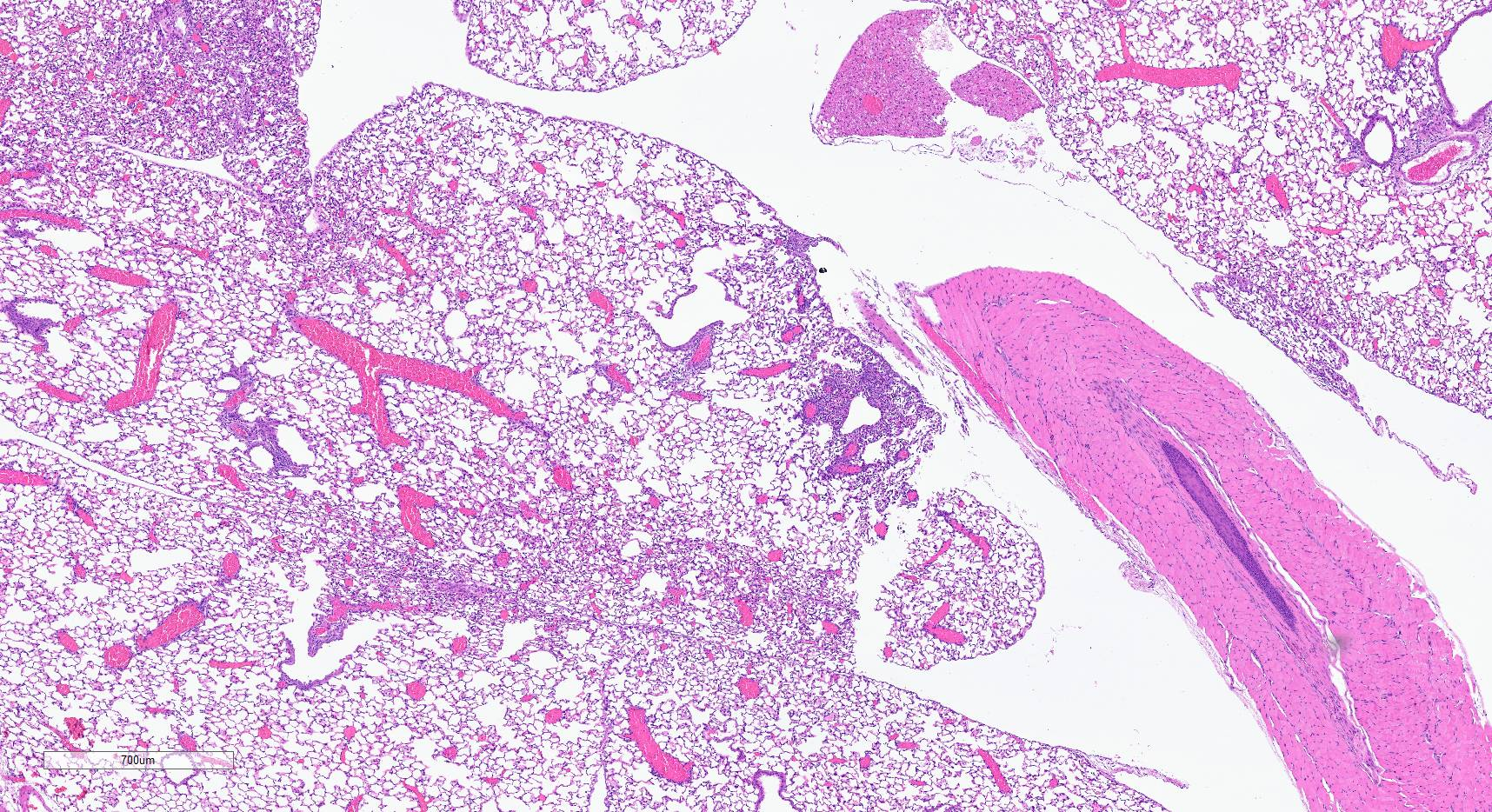

Supplement: S1 Raw Images — (ZIP) [file ppat.1014167.s002.zip › Fig.8A aged LAV.tif]

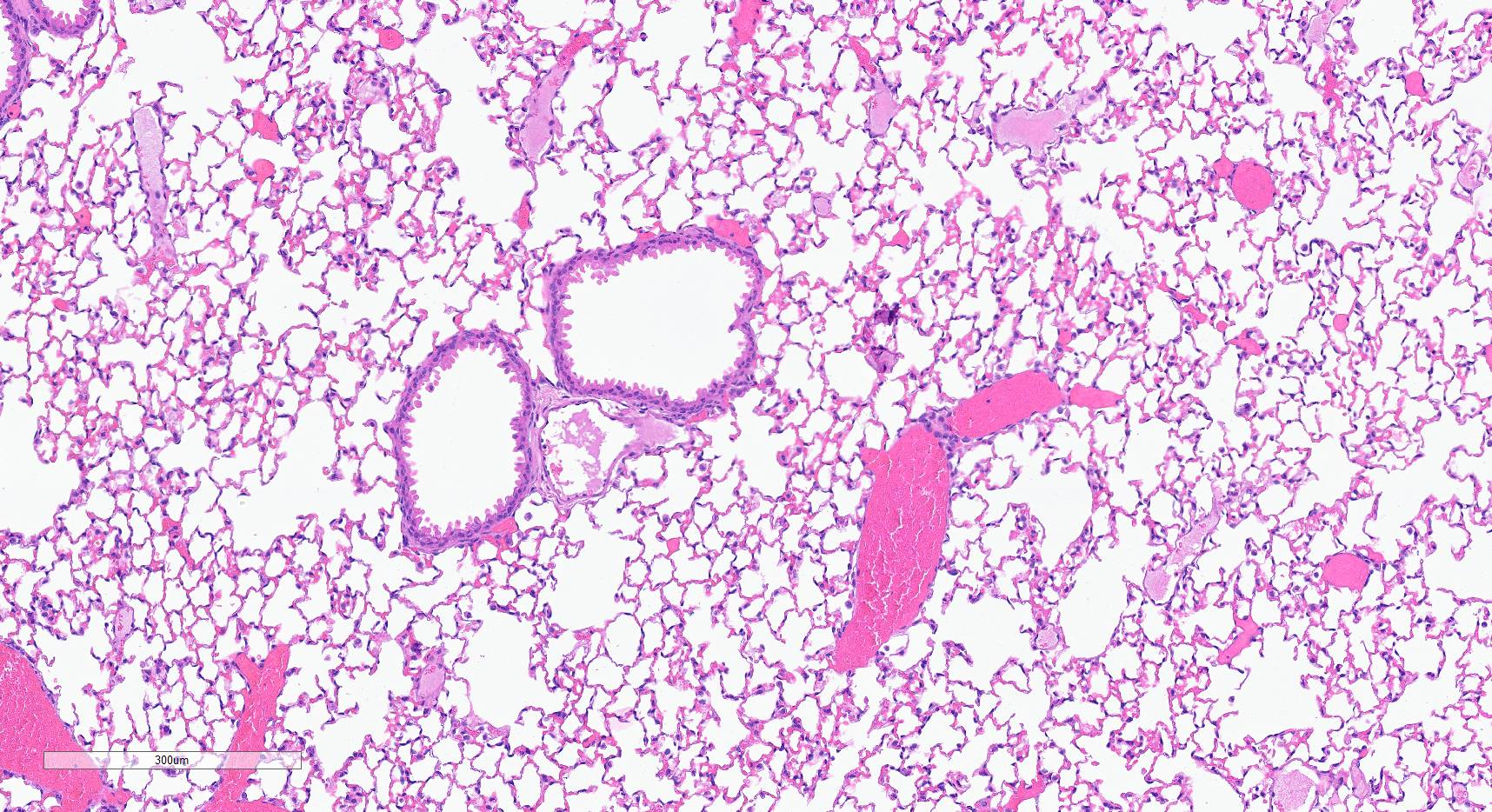

Supplement: S1 Raw Images — (ZIP) [file ppat.1014167.s002.zip › Fig.8A aged naive zoom in .tif]

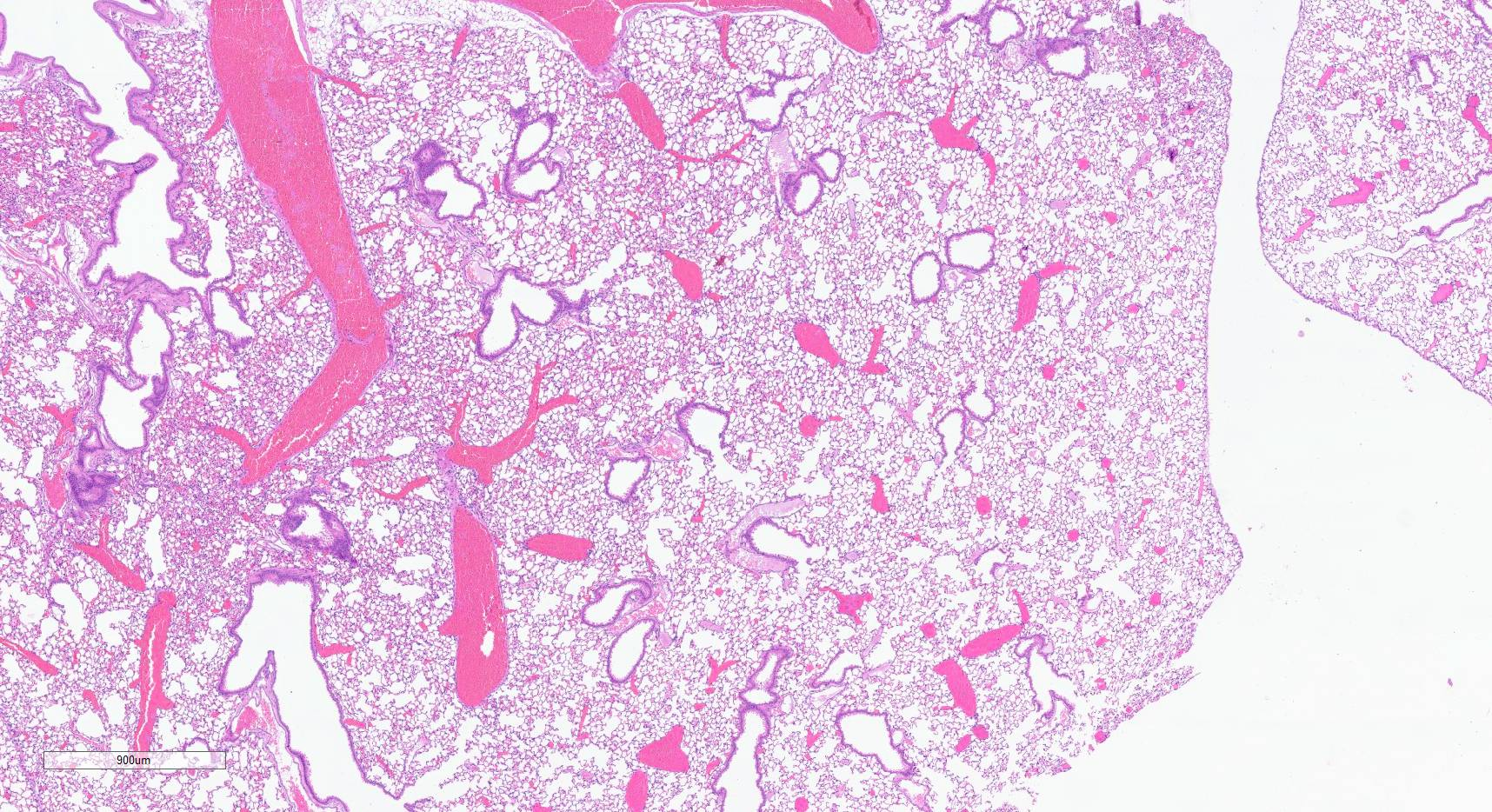

Supplement: S1 Raw Images — (ZIP) [file ppat.1014167.s002.zip › Fig.8A aged naive.tif]

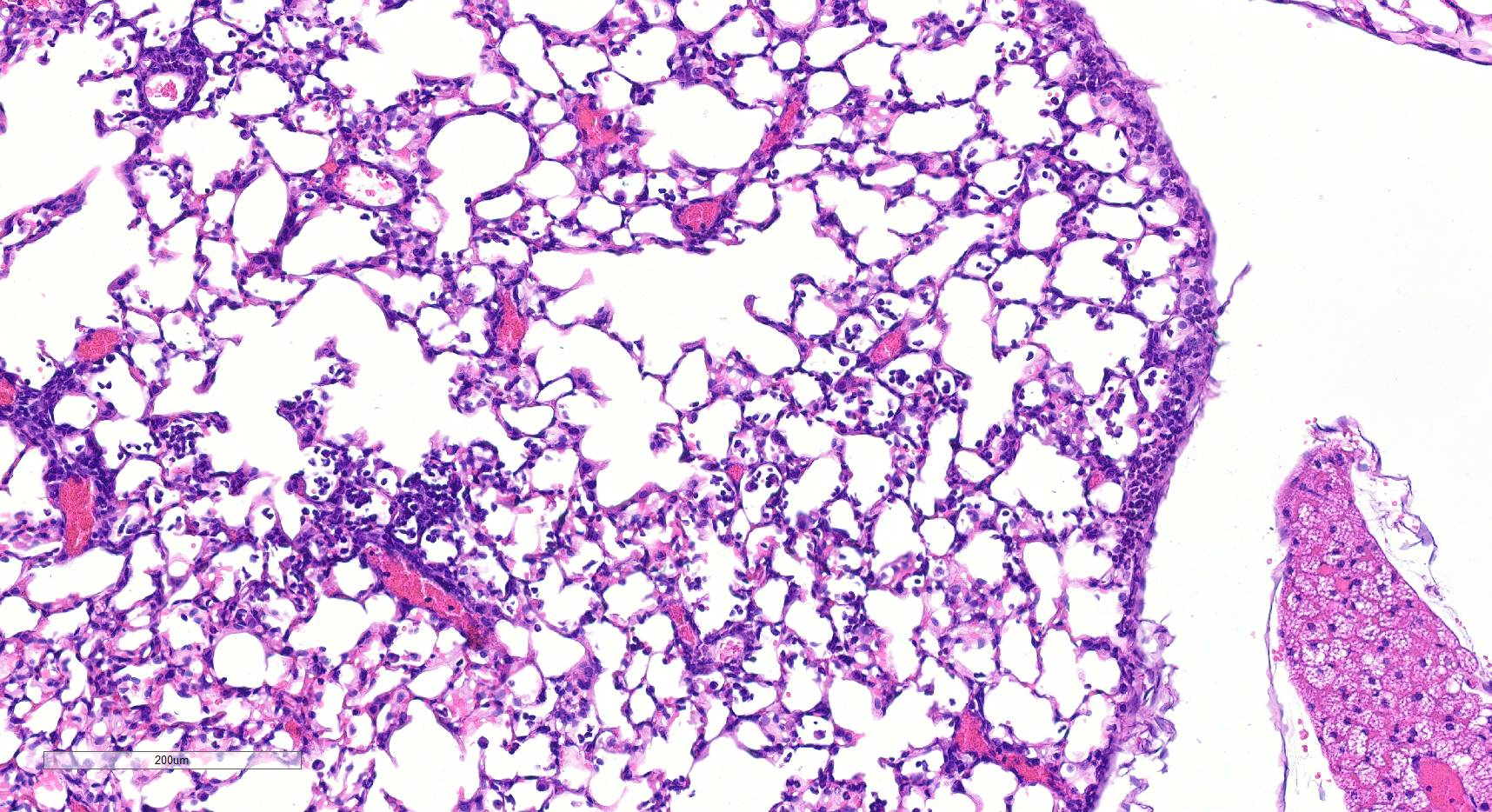

Supplement: S1 Raw Images — (ZIP) [file ppat.1014167.s002.zip › Fig.8A aged WT zoom in.tif]

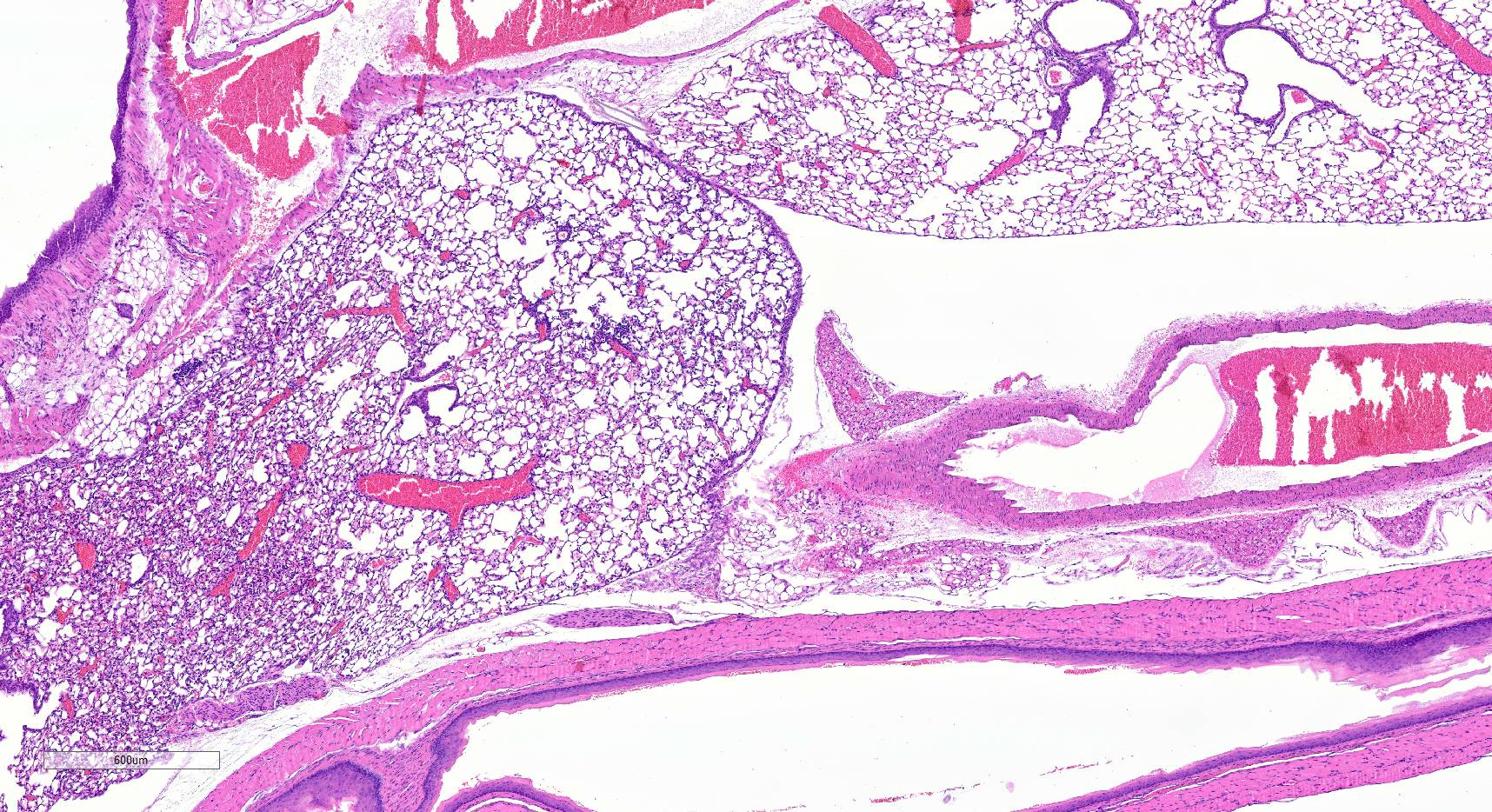

Supplement: S1 Raw Images — (ZIP) [file ppat.1014167.s002.zip › Fig.8A aged WT.tif]

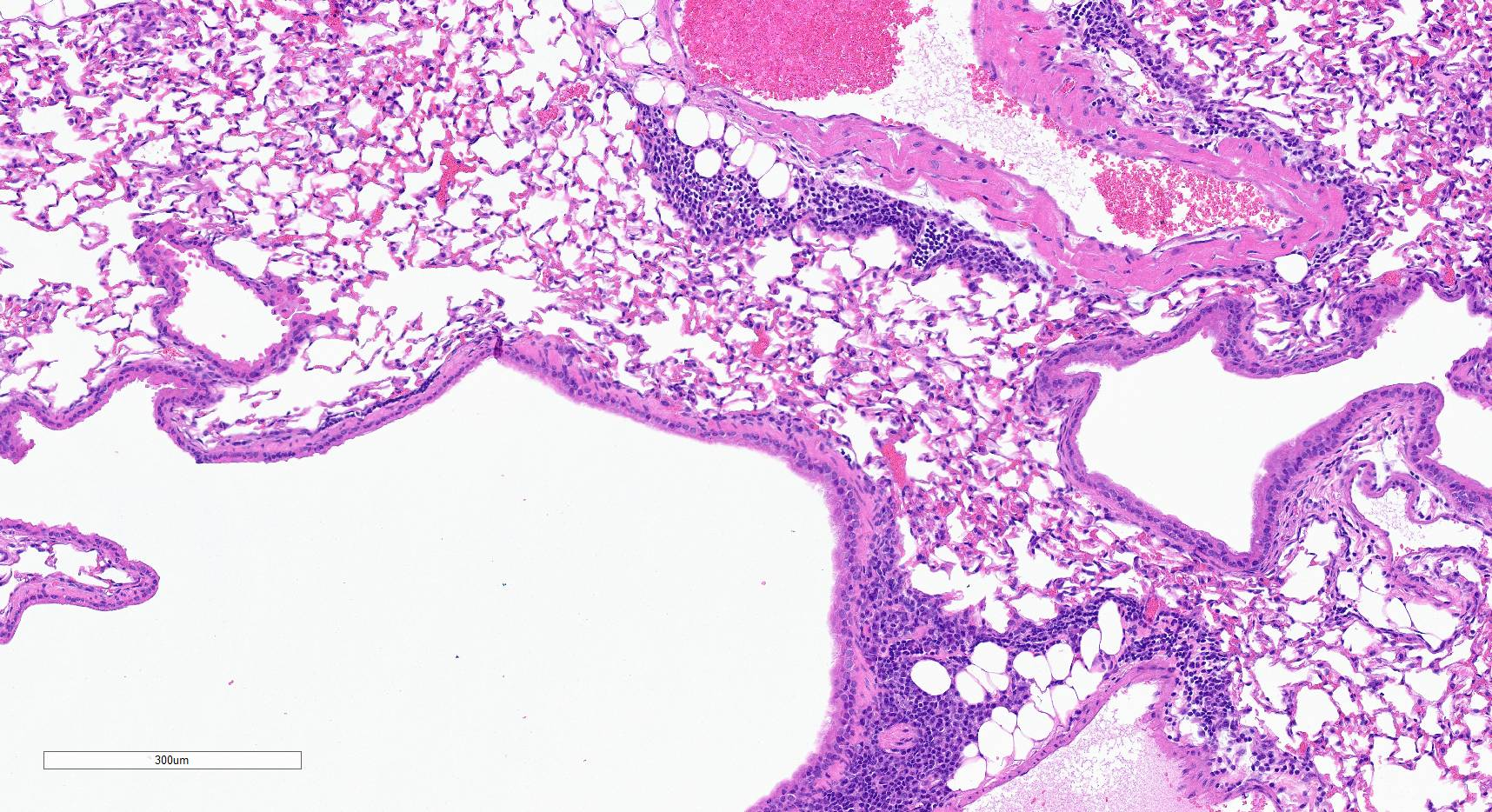

Supplement: S1 Raw Images — (ZIP) [file ppat.1014167.s002.zip › Fig.8A young LAV zoom in .tif]

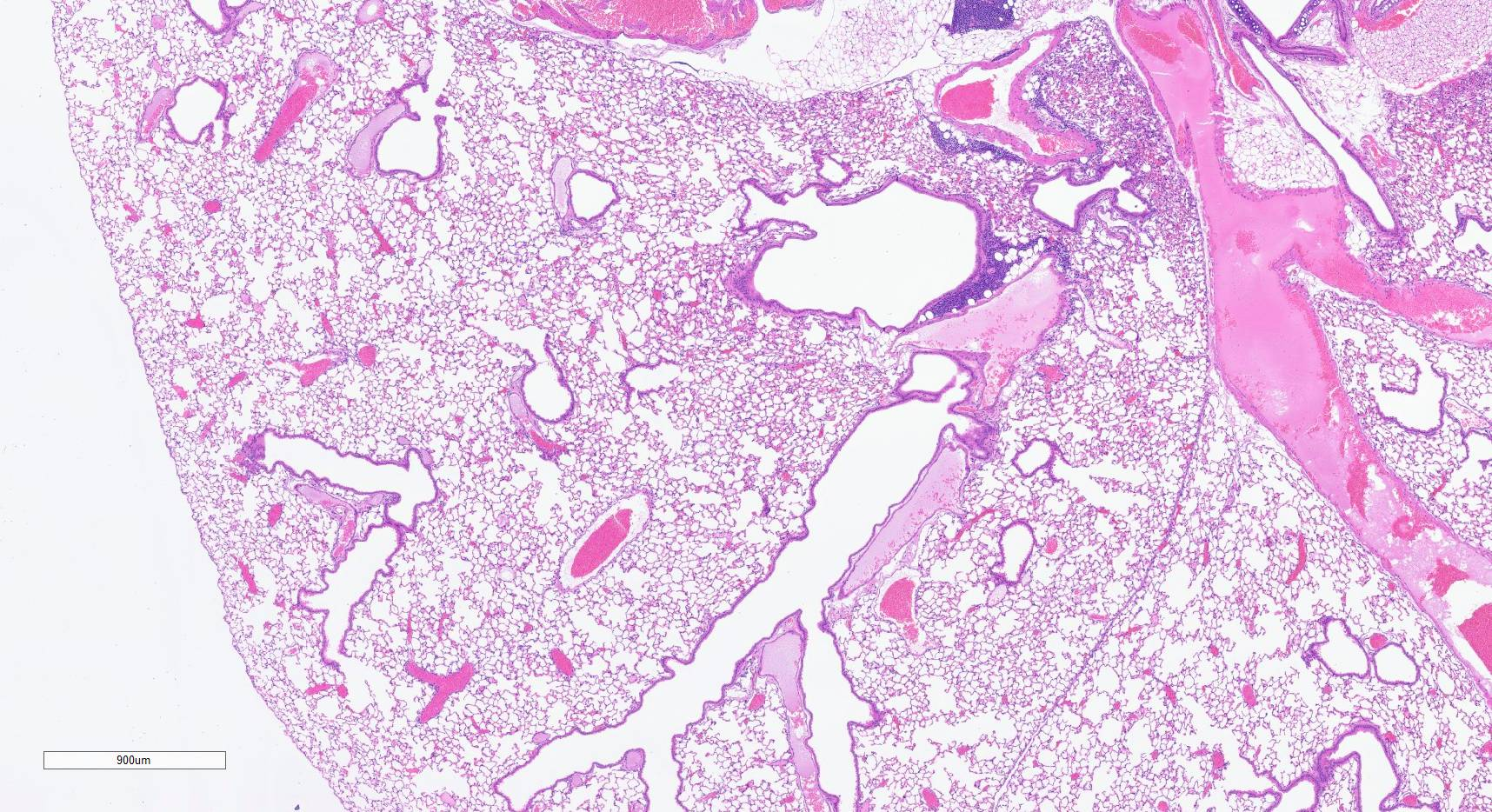

Supplement: S1 Raw Images — (ZIP) [file ppat.1014167.s002.zip › Fig.8A young LAV.tif]

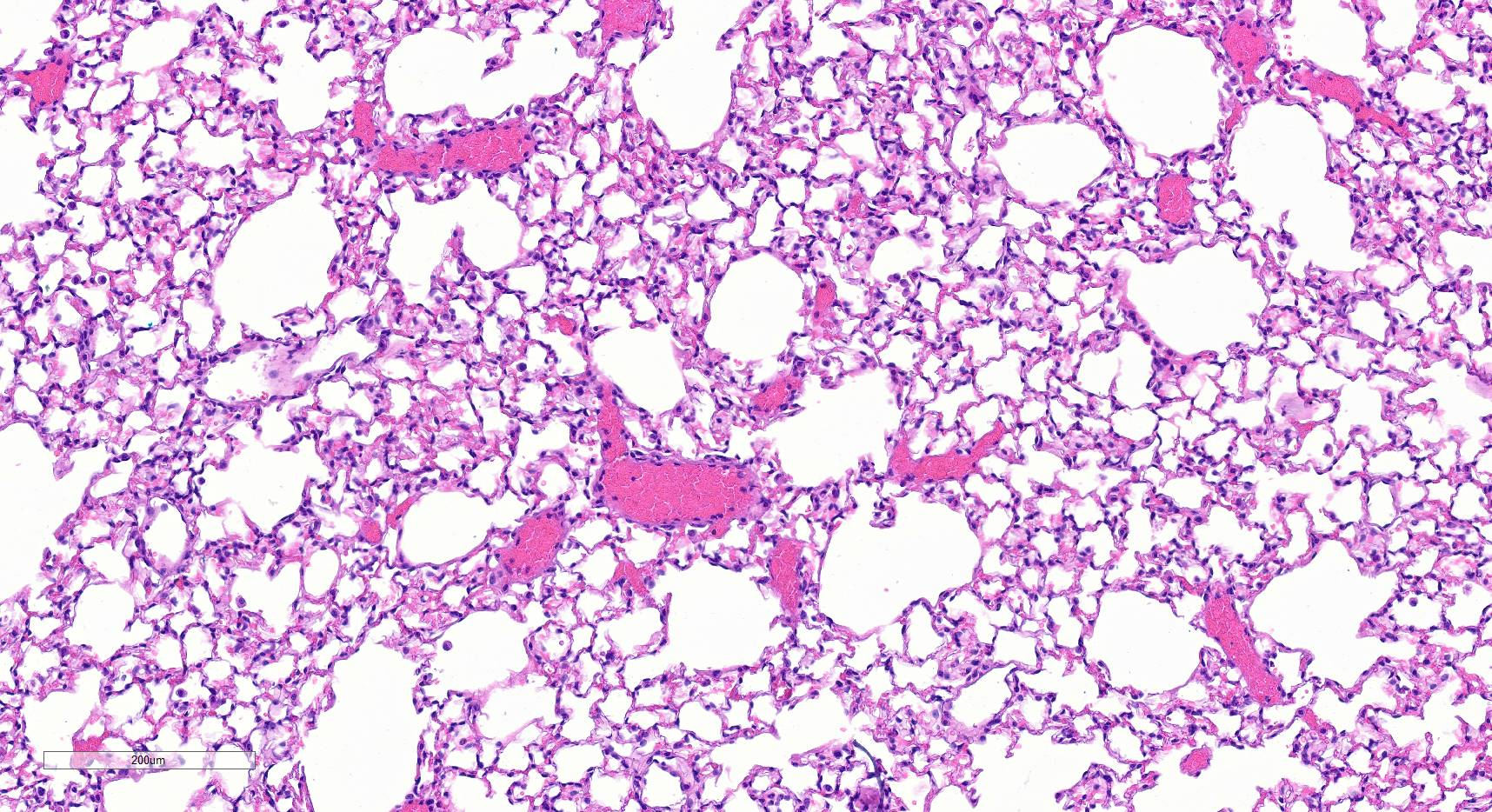

Supplement: S1 Raw Images — (ZIP) [file ppat.1014167.s002.zip › Fig.8A young naive zoom in.tif]

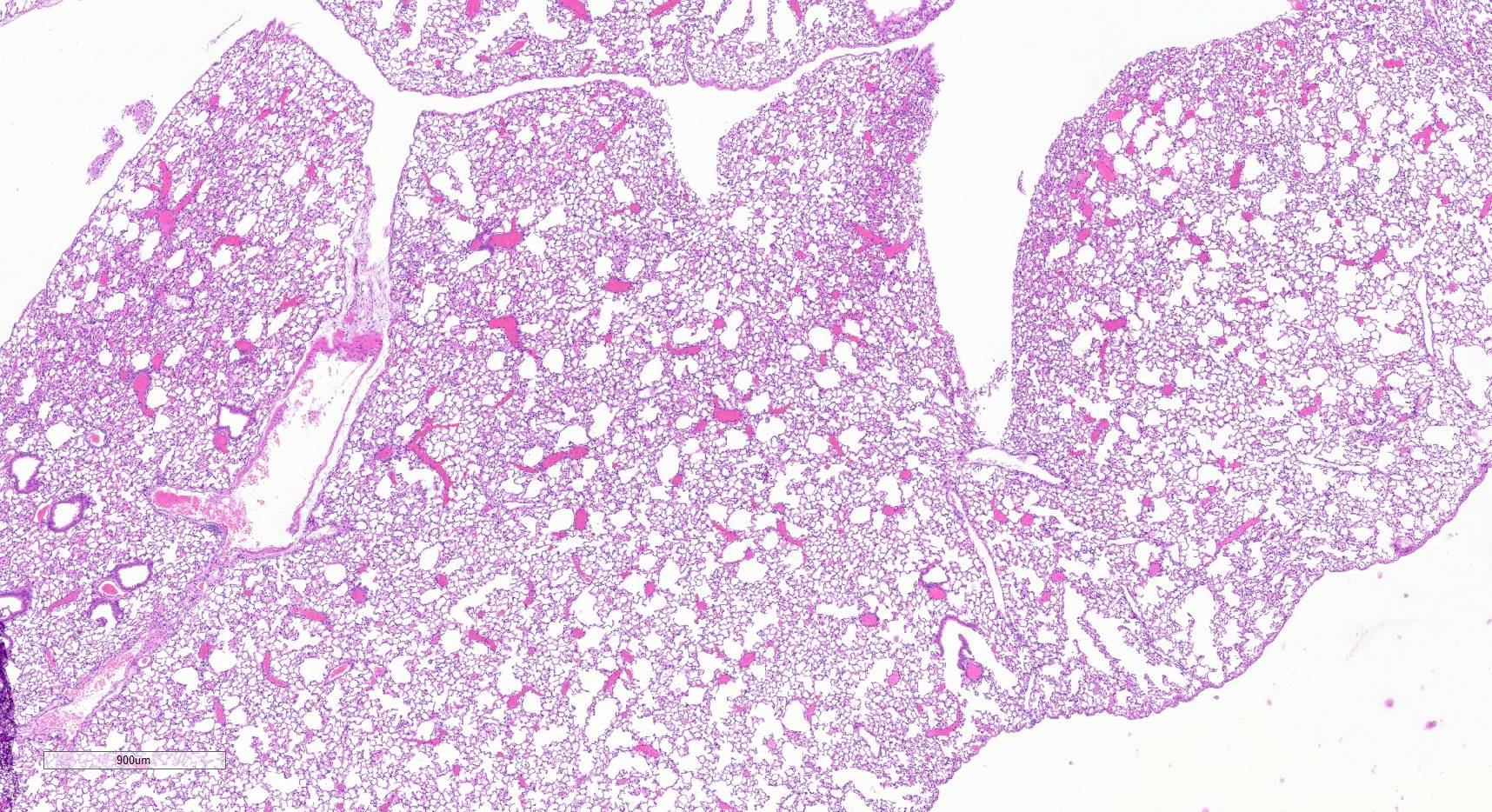

Supplement: S1 Raw Images — (ZIP) [file ppat.1014167.s002.zip › Fig.8A young naive.tif]

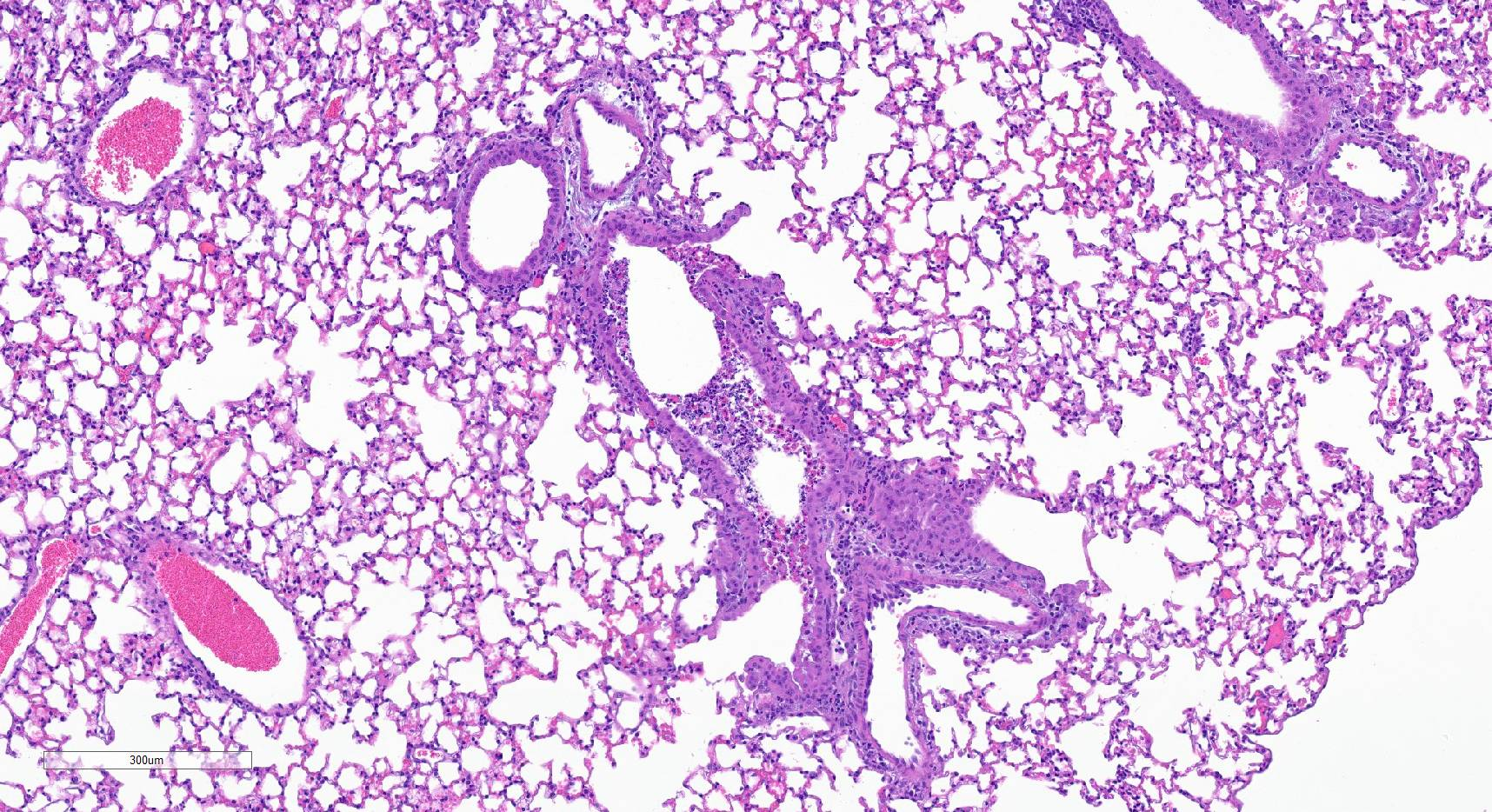

Supplement: S1 Raw Images — (ZIP) [file ppat.1014167.s002.zip › Fig.8A young WT zoom in.tif]

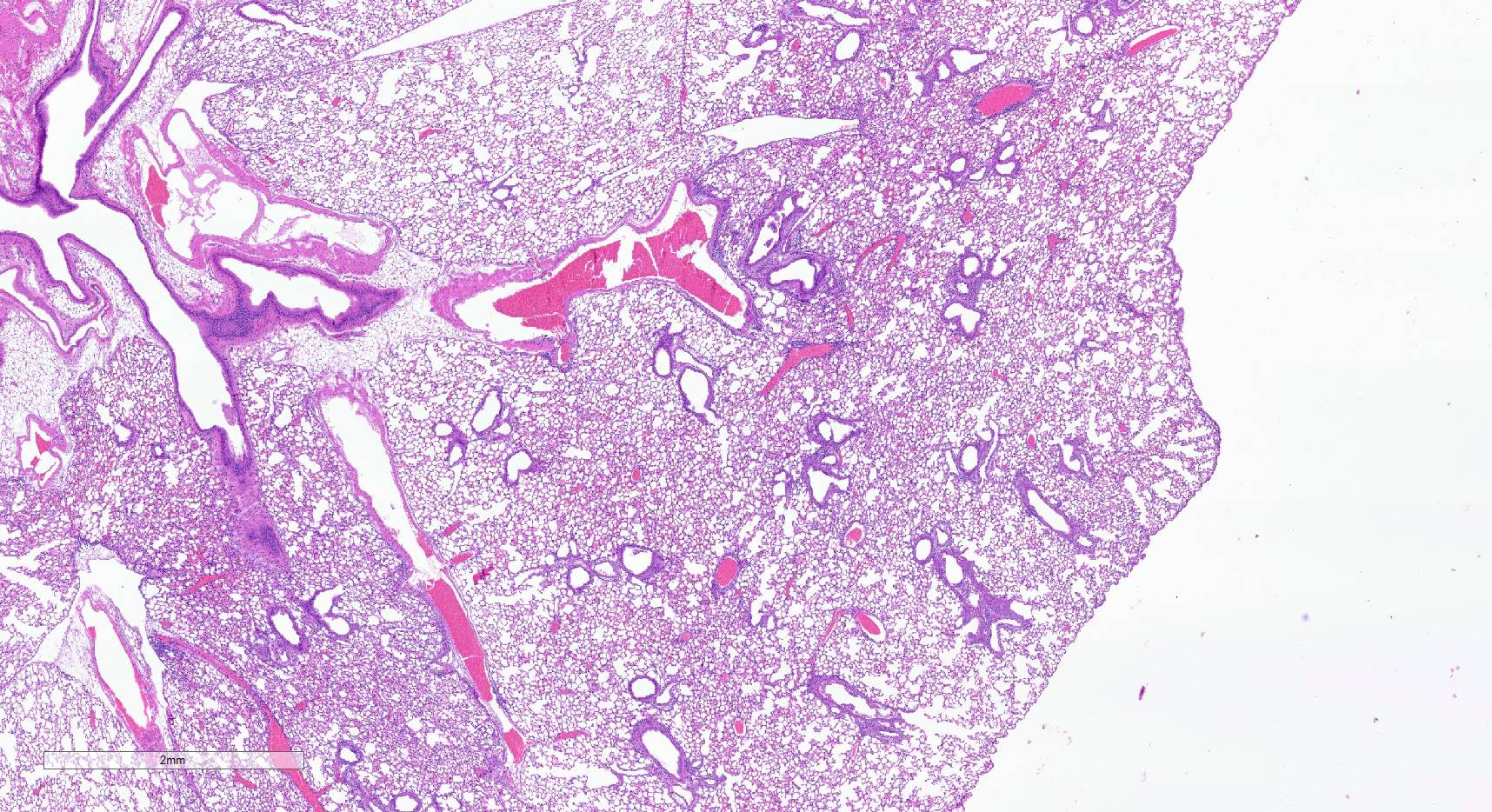

Supplement: S1 Raw Images — (ZIP) [file ppat.1014167.s002.zip › Fig.8A young WT.tif]

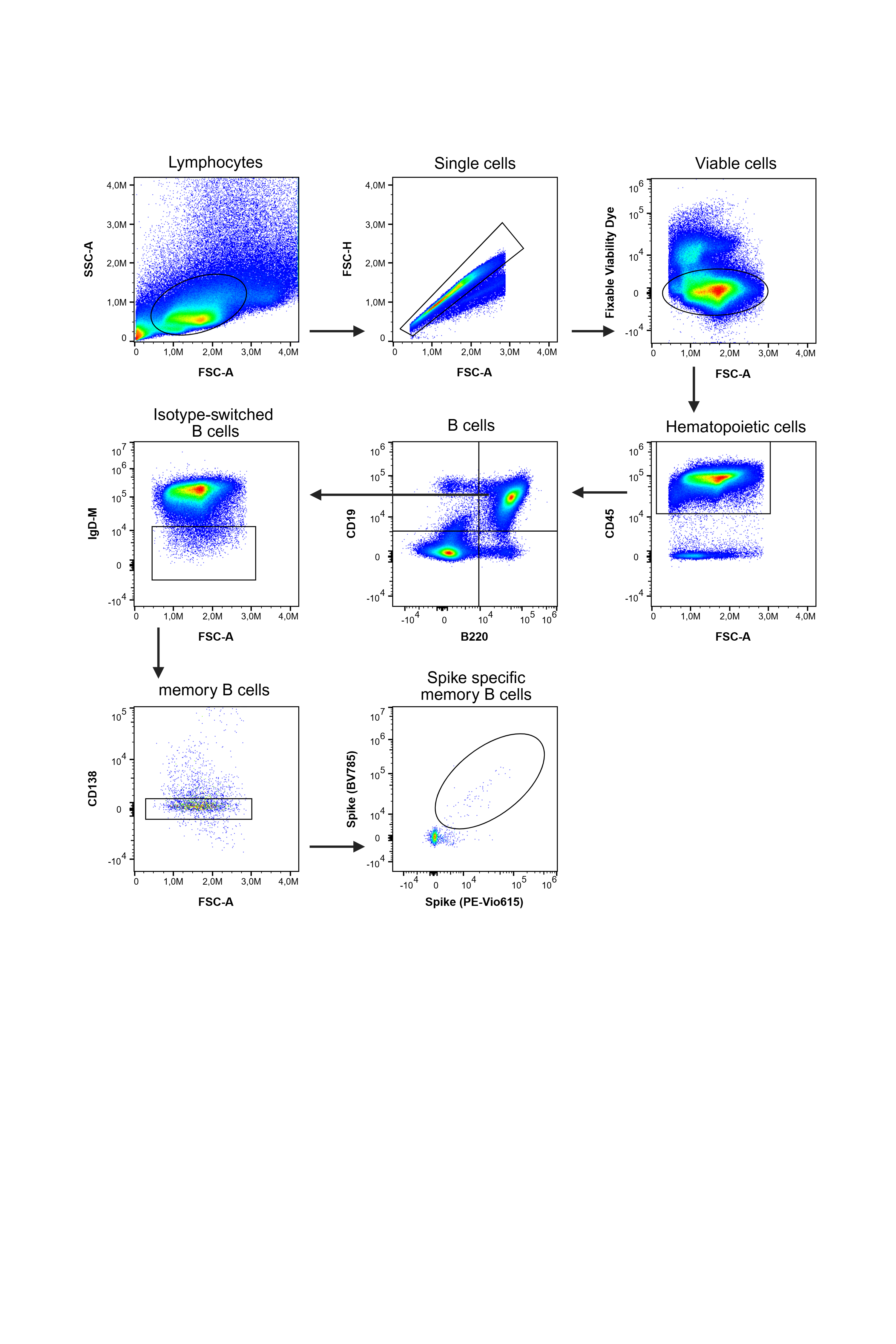

Supplement: S5 Fig — (TIF) [file ppat.1014167.s007.tif]

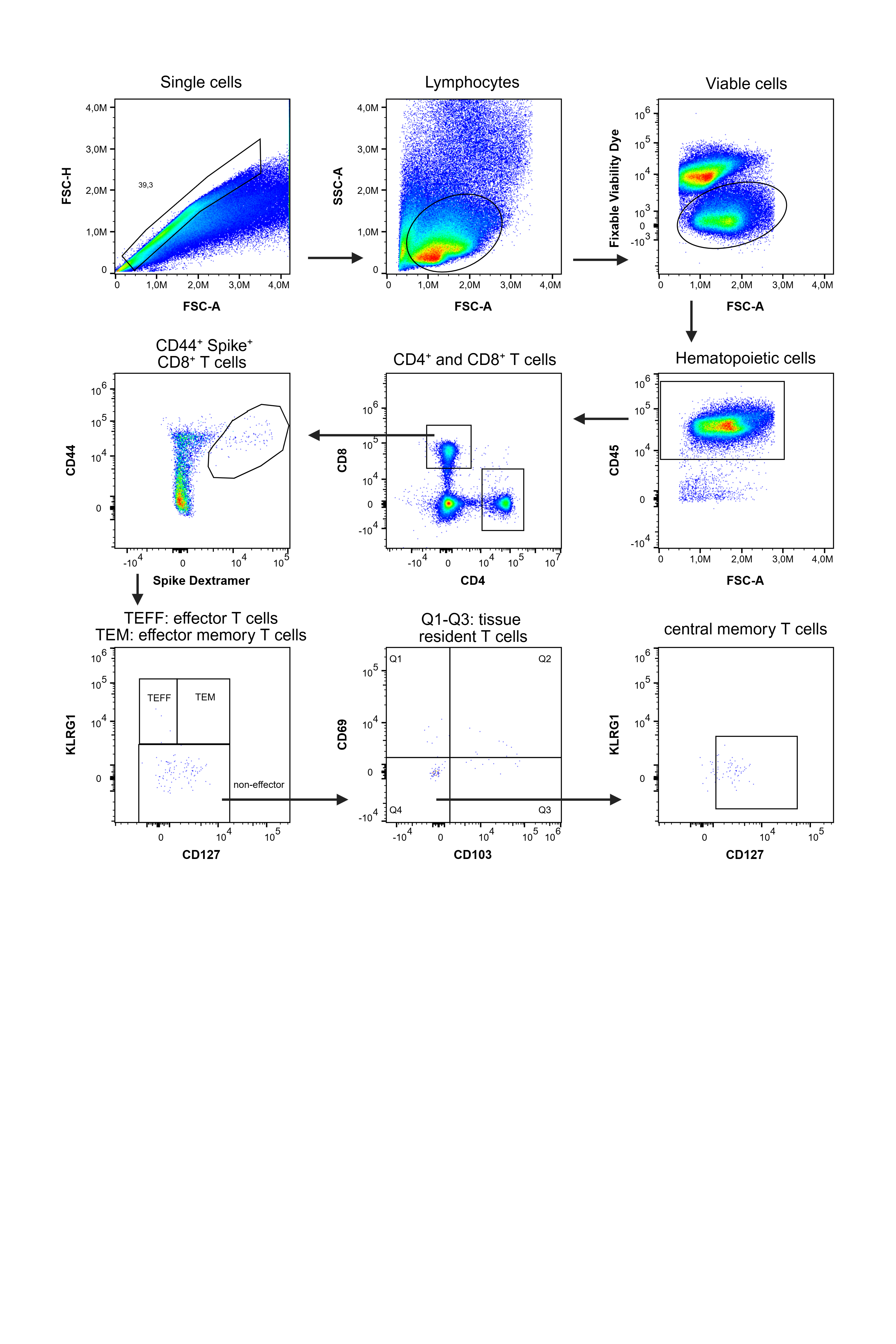

Supplement: S6 Fig — (TIF) [file ppat.1014167.s008.tif]

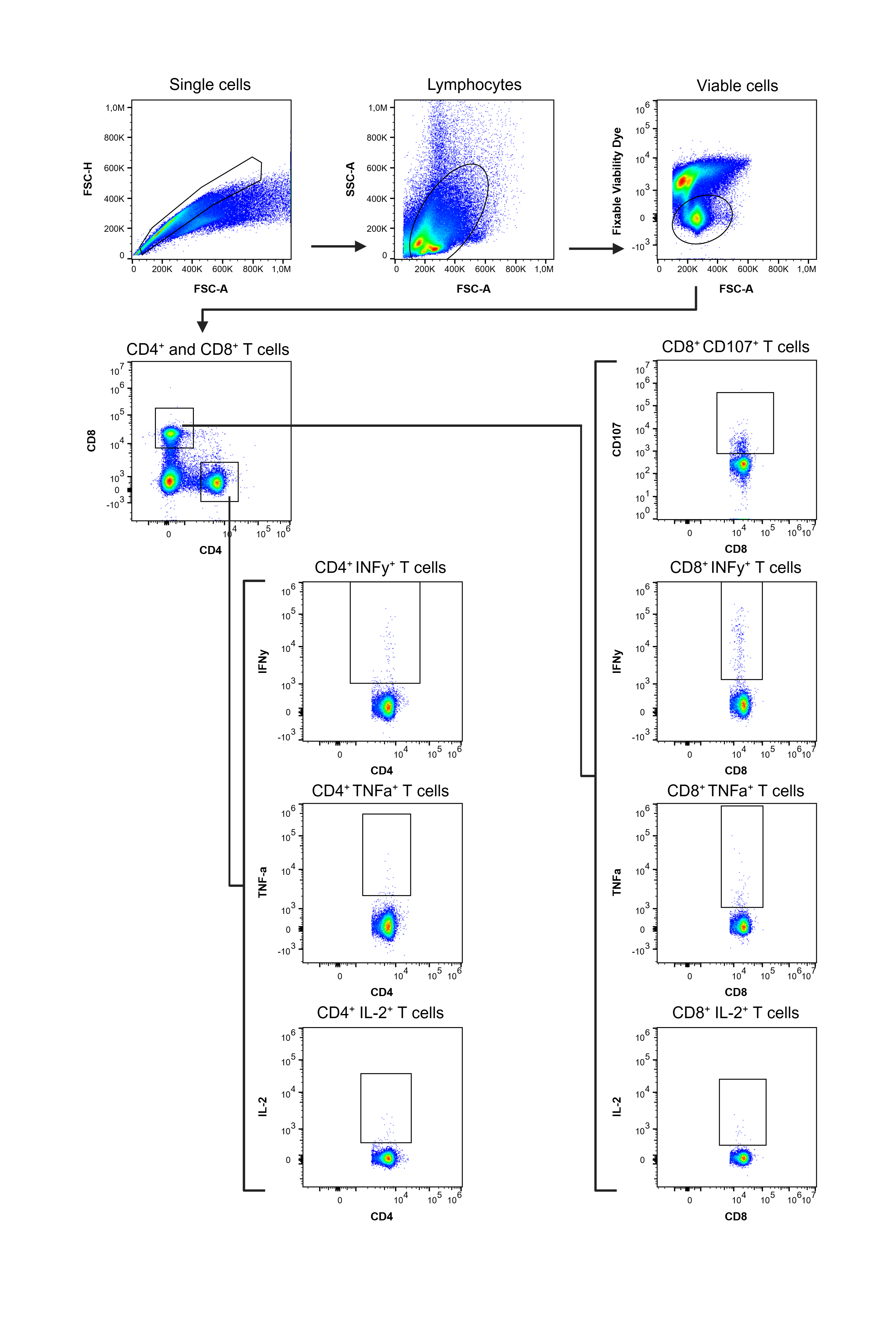

Supplement: S7 Fig — (TIF) [file ppat.1014167.s009.tif]
