# Supplementary material for: Live attenuated vaccination protects aged chimeric ACE2 mice from severe SARS-CoV-2 pathogenicity in vivo
Source: PLoS Pathog. 2026 Apr 22;22(4):e1014167. doi: 10.1371/journal.ppat.1014167 (PMC13132463; doi:10.1371/journal.ppat.1014167)
Supplement: Attachment — Submitted filename: PPATHOGENS-D-25-02404_Reviewer.pdf [file ppat.1014167.s010.pdf]

Reviewer Recommendation and Comments for Manuscript Number PPATHOGENS-D-25-02404

REVIEWER COMMENTS

Reviewer #1:

The manuscript "***Live attenuated vaccination protects aged chACE2 mice from severe SARS-Co-2 pathogenicity in vivo"*** by Russ et al., introduces a novel murine model (chACE2) that expresses a human-mouse chimeric ACE2 receptor at physiological levels, enabling SARS-CoV-2 infection without the neuroinvasive lethality seen in previous models. Using this model, the authors evaluate a live attenuated vaccine candidate lacking Nsp16 methyltransferase activity (LAV^{Nsp16}), demonstrating its ability to elicits mucosal and systemic immunity and confer protection against severe disease in aged mice challenged with both homologous and heterologous SARS-CoV-2 variants.

The manuscript is well-conceived and contributes to the field. With revisions addressing the points, it will be suitable for publication in *Plos Pathogens*. The following points need to be addressed by the authors:

(i) Abstract: it clearly identifies the problem (lack of physiologically relevant mouse models for SARS-CoV-2, especially in aged populations) and introduces a novel solution (the chACE2 mouse model). The structure of the abstract is logical, moving from model development to vaccine testing and outcomes. Areas for improvement:
- Overuse of general statements. Phrases like "Strikingly..." and "Together, we show..." are more appropriate for the discussion; the abstract should remain objective and concise.

(ii) Introduction: it provides an overview of the COVID-19 vaccine landscape; it identifies the limitations of current murine models (the need for improved systems to study age-related SARS-CoV-2 pathology); and it introduces the rationale for targeting Nsp16 and the development LAVNsp16. However, some aspects can be improved:
- Clarify claims such as "problems of vaccine strategies targeting solely the S protein became visible" is vague. Specify what problems (immune escape, reduced efficacy, ...).
- "huge number of sub lineages" is informal. Consider "extensive diversification of Omicron sublineages".
- The introduction refers on age-related immune decline but could benefit from a mechanistic discussion such as immunosenescence or impaired IFN signaling (1-2 sentences).

- What advantages does the chimera offer vs full human ACE2? Is this discussed in the Discussion section? Not clear in the introduction as "model justification".

(iii) Results: this section presents promising data on the attenuation and immunogenicity of LAV^{Nsp16} and the utility of the chACE2 model. However, the conclusions would be strengthened by addressing the limitations mentioned below.

- The statement in lines 131-132 is not entirely accurate, as wild-type mice have been shown to be susceptible to infection with certain SARS-CoV-2 variants, including B.1.351/Beta and BA.1.1/Omicron, as reported by Tarrés-Freixas et al. (Front Microbiol. 2022 May 4;13:840757. doi: 10.3389/fmicb.2022.840757. eCollection 2022).
- Line 137: please replace "SARS-CoV-1" with "SARS-CoV", as the correct nomenclature for the virus identified in 2002-2003 is "SARS-CoV" (https://ictv.global/report/chapter/coronaviridae/coronaviridae/betacoronavirus)
- Line 141: please replace "qRT-PCR" with the correct terminology "RT-qPCR", which is the standard abbreviation for revers transcription quantitative PCR.
- Line 142-143: the statement regarding enhanced tissue damaged observed in the BAL is misleading, as BAL is a fluid sample. While the figure legend notes that protein concentration in BAL was used as a surrogate marker for tissue injury, this rationale should also be explicitly stated in the results section. Additionally, it would be important to clarify why histopathological analysis of lung tissue was not performed to directly evaluate tissue damage.
- Only viral RNA quantification has been performed; was infectious virus titration (e.g., plaque assay or $TCID_{50}$) conducted to confirm the presence of replication-competent virus?
- Figure 1E: is there any data available on the progression of disease in this animal model beyond 7 dpi with LAV^{Nsp16}? Specifically, could the onset or severity of disease be delayed?
- Although the authors demonstrate that chACE2 mice support replication of both wt SARS-CoV-2 and the attenuated strain (Line 179), with clear differences in pathogenicity (absence of neuroinvasion and brain viral RNA), the authors rely on viral RNA quantification. It is not clear whether infectious virus titration was performed which is critical to confirm active replication and/or attenuation.
- Figure 3E interpretation: the absence of viral RNA in the brain is crucial, but histopathological confirmation would be valuable to rule out subclinical neuroinflammation.
- The authors provide flow cytometry data showing innate immune cell infiltration and RT-qPCR data supporting local immune activation, but this reviewer questions the rationale for using RT-qPCR to assess IFNbeta and ISGs

expression instead of flow cytometry to measure protein, which would provide more direct evidence of cytokine production and cellular activation.

- The authors report anti-S IgG levels (Line 211-234) but do not assess neutralizing capacity, which is the gold standard for measuring neutralizing antibodies, thus, the ability to block virus infection.
- Also, for aged mice (Lines 235-263) histological analysis of lung will support the claim of reduced tissue damage in those infected with LAV^{Nsp16}.
- The reduced ISG induction and immune cell infiltration are consistent with attenuation. However, age-related differences in baseline immune status should be discussed.
- Again, in the section "homologous or heterologous SARS-CoV-2 challenge" (Lines 265-284): viral replication in BAL and lungs was assessed via RT-qPCR and no infectious titers are provided. Quantifying replication-competent virus is essential to validate vaccine efficacy.
- The Delta variant challenge is described as resulting in no disease within four days, suggesting slower replication, but this interpretation requires caution. Longer periods post-challenge (7-10dpi) would be necessary to fully assess pathogenicity and vaccine protection.

(iv) Discussion:

- While live attenuated vaccines are known for their efficacy, the manuscript should more explicitly address the limitations of this approach, particularly in immunocompromised populations, including the elderly. This is especially relevant given the focus on aged mice.
- The authors report strong mucosa and systemic immunity, but further elaboration on the specific immune parameters measured would be beneficial.
- The manuscript would benefit from a more detailed discussion of the heterologous variants used, including their relevance to circulating strains and implications for cross-protection.

Overall assessment: the results section presents compelling data supporting the utility of the chACE2 model and the immunogenicity and safety of LAV^{Nsp16}. However, several key aspects mentioned above require clarification or additional information: (i) infectious virus titration, (ii) histopathological assessment of tissue damage, and (iii) assessment of neutralizing antibody responses.

(v) Materials and methods:

- This section would benefit from a thorough revision and the inclusion of more detailed and specific methodological information to improve clarity and reproducibility. For instance, the description in lines 399-402 is difficult to follow: "Eight-week-old K18-hACE2 mice (B6.Cg-Tg(K18-ACE2)2Prlmn/J) were infected with 1000 plaque-forming units (PFU) of virus in 30 µl PBS. Young (8 to 12 weeks) and aged (40 to 42 weeks) chACE2 mice (C57BL/6-Ace2-tm1(ACE2*1)-TW) received 7 x104 PFU in 30 µl PBS". The protocol should be described more precisely, it is confusing.

(vi) Figures:

- Figure 1 legend: please specific the virus more clearly by replacing “1,000PFU of wt” with “1,000 PFU of SARS-CoV-2 wt”.

(vii) Revise grammar and style, for example:

- “did not result in diseases” should be revised to “did not result in clinical disease”.
- “suggesting rather slow replication kinetics” consider rephrasing to “suggesting delayed replication kinetics”.
- or, “Fig. 3. Chimeric ACE2 supports SARS-CoV-2…” replace it by “Fig. 3. Chimeric ACE2 mice supports SARS-CoV-2 wt ..:”

I recommend major revision to address these points and strengthen the manuscript’s scientific rigor.
