# Supplementary material for: Live attenuated vaccination protects aged chimeric ACE2 mice from severe SARS-CoV-2 pathogenicity in vivo
Source: PLoS Pathog. 2026 Apr 22;22(4):e1014167. doi: 10.1371/journal.ppat.1014167 (PMC13132463; doi:10.1371/journal.ppat.1014167)
Supplement: Attachment — Submitted filename: Point to point Response.pdf [file ppat.1014167.s012.pdf]

Point to point

Major

Reviewer #1

(i) infectious virus titration

We thank the reviewer for the valid point. We measured infectious virus titer exemplary in aged mice and compared it to genome titers **(new Figure 6C and 6D).** As expected, we found that a lower infectious titer compared to genome titer for both viruses, reflecting the general accepted idea that only a fraction of released virus is infectious. Importantly, we were able to detect +/- 10e4 infectious viral particles in the lung and BAL of infected mice, with reduced levels of the attenuated LAV.

(ii) histopathological assessment of tissue damage

In addition to the BSA assay analyzing epithelial barrier function, we now included histopathological assessment of lung damage upon infection **(new Fig 8)**.

(*iii) assessment of neutralizing antibody responses.*

The reviewer is correct, this has been missing. We now included the analysis of the neutralizing Ab response in **new Fig. 5E**.

Reviewer #2

1. Sequencing of SARS-CoV-2 recovered from infected chACE2 mice should be performed to exclude the generation of a chACE2-adapted virus. This could limit the model´s utility for pathogenesis studies, though it would still be valid for evaluating vaccines or antivirals.

Very interesting point. We performed NGS RNA Seq to analyze replicating wt virus in chACE2 and K18-hACE2 mice fur days postinfection. We did not find any (mouse) adaptations in the RBD domain of Spike, as seen for example in mouse-adapted strains such as MA20. **New Supplementary Figure 2** and **text line 199-201**.

2. To study the viral growth in the chACE2 mouse model, viral RNA was quantified by RT-qPCR (Figs. 3, 6 and 8). To enhance physiological relevance, infectious virus titers should be provided.

We thank the reviewer for the valid point. We measured infectious virus titer exemplary in aged mice and compared it to genome titers **(New Figure 6C and 6D, new Supplement Figure 3).** As expected, we found an about three log lower infectious titer compared to genome titer, reflecting the general accepted idea that only a fraction of released virus is

Universitätsklinikum Erlangen
Anstalt des öffentlichen Rechts
Aufsichtsrat (Vorsitzender):
Staatsminister Markus Blume
Telefon: +49 9131 85-0
Fax: +49 9131 85-36783
www.uk-erlangen.de

Harald-zur-Hausen-Institut für Virologie
Schlossgarten 4
91054 Erlangen
Telefon: +49 9131 85-32035
Fax: +49 9131 85-32101
www.virologie.uk-erlangen.de

Stadt- und Kreissparkasse Erlangen
Höchstadt Herzogenaurach
IBAN: DE28 7635 0000 0000 0004 64
BIC: BYLADEM1ERH
Steuer-Nr. 216/114/80005
USt-ID-Nr. DE 248558812

Friedrich-Alexander-Universität
Medizinische Fakultät

KOM/04-2025

infectious. Importantly, we were able to detect +/- 10^4 infectious viral particles in the lung and BAL of infected mice, with reduced levels of the attenuated LAV.

3. FIg. 4. Are the differences in immune cell infiltration between wt and LAVnsp16 infection statistically significant?

We reanalyzed the immune cell infiltration in the lungs of young and aged mice and included statistics. Especially in young mice, immune cell infiltration upon LAV infection is less prominent compared to wt infection, especially for monocytes for neutrophils, most likely due to the attenuated nature of the virus. The difference between LAV and WT is not as clear in aged mice, maybe due to a less efficient antiviral activity in aged mice **(New Figures 4A-E and 7A-E).**

4. No significant differences in infiltrating cells and ISG expression are observed between LAVnsp16 and SARS-CoV-2 wt at 4 and 7 dpi in K18-hACE2 mice (Fig. 4). Similarly, LAVnsp16 and SARS-CoV-2 wt also induced comparable cell infiltration and tissue damage (Fig. 7) in aged chACE2 mice. These results suggest residual virulence in LAVnsp16 and additional attenuation may be needed for further vaccine development. Could the authors discuss this point?

For efficient antiviral immunity, LAV designs need to induce local innate immune responses. Thus, immunogenicity of LAV Nsp16 in chACE2 mice depicted in Fig 4 and 7 suggests the efficient mounting of such a response. With regard to safety of that innate response, in young animals, we see decreased immune cell infiltration (Mono, Neutrophils and NK cells,) and ISG induction upon LAV infection compared to wt infection **(New Figure 4).** The strong attenuation of LAV-Nsp16 shows especially in aged mice. Prominent are here the low clinal score (Fig. **6A, B, 6E, 6F**), the strongly reduced viral load 4 dpi (Fig. **6D**), as well as in the strongly reduced lung tissue damage seen in aged mice (Fig. **8C**). Nevertheless, we will combine Nsp16 with additional mutations in the future to further attenuate the current LAV design. **Mentioned in line 415-417.**

5. Fig. 5B. Please discuss the observation that the anti-S IgG response induced by LAVnsp16 in BAL is not superior to naive mice, despite intranasal administration and high LAV RNA levels in BAL and lungs.

We thank the reviewer for spotting this. Indeed, we identified a technical problem in the FACS staining of two naïve animals (Fig. 5B). Thus, we re-analyzed the data (IgG level in BAL and sera) using ELISA. Similar to the IgG levels found in serum (new Fig. 5A), we now see solid IgG levels upon infection with wt and LAV (p=0.06) in the BAL of the animals

(new Fig.5 B). We suspect the attenuated nature of the LAVs as well as the potent control of infection, especially in young mice, as possible reasons for the lower IgG levels compared to wt. In the future, we will use increasing amounts of inoculate to test whether a stronger local antibody concentration can be induced.

6. Fig. 6G. Different ACE-2 protein bands patterns are observed between young and aged mice in both wt B6 and in chACE2 models. Please discuss.

Similar to humans, two different isoforms of murine ACE2 are expressed (PMID: 17340620) and is posttranslationally modified (PMID: 38063421). Thus, it is very conceivable that either an age-dependent change in isoform expression or a posttranslational modification (such as glycosylation) results in the shift towards the upper of the two main mACE2 bands visible in **Fig 6H.** A similar age dependent shift for murine ACE2 expression has been published previously (PMID: 34774871). Together with the stronger expression of ACE2 in aged mice **(Fig. 6H),** this change might very well be implicated in the enhanced viral replication and pathogenicity in aged mice. We discussed this possibility briefly in **line 385-389.**

7. Fig. 1D and Fig. 7F. Protein concentration in BAL fluid is used as a surrogate for tissue damage. This indirect measure should be complemented or replaced by histopathological analysis to confirm vaccine attenuation in both in young and aged mice.

Protein concentration as surrogate for lung epithelial barrier and tissue damage is an established method and frequently used (for example in PMID: **21094033 or PMID: 40017475**). In addition, we complemented the protein content data with histopathological analysis for WT and LAV infection of young mice to assess inflammatory events in the lung **(new Fig. 8**).

8. Fig. 8B and 8D show that the heterologous Delta variant challenge in non-immunized animals does not cause significant weight loss or mortality, suggesting lower replication efficiency in chACE2 mice compared to B.1 variant. The conclusion that immunization potently protects against heterologous and homologous SARS-CoV-2 replication (line 283) should be revised.

The reviewer is right. We rewrote the passage in question **(line 305).**

Reviewer #3

A further characterization of actual induced pathology or lack thereof would benefit this initial model characterization. Currently analysis focusses on viral genome quantification (no infectious virus), mortality and immune response. Especially if consequences for elderly shall be mimicked and side effects of LAV shall be evaluated, further analysis of infectious virus titers and pathological analysis (at least viral antigen quantification in respiratory tissue) would be of added value. Also a comparison of infected cell types vs human cases would be interesting if this manuscript is the first who describes this model. Therefore, I recommend follow-up analysis of tissues in regards of histopathological analysis.

The reviewer has a good point. We therefore now compare viral genome titers with infectious titer **(new Fig 6C, CD)** and added a histological analysis of wt and LAV infected lung tissue of aged mice **(new Fig 8).**

For characterization of reduced pathology of the LAV in K18-ACE2 mice the authors still observe high viral genome in the brain of 50% of animals. Yet mortality is reduced. Can the authors explain that? Also here, histopathology or infectious virus titration would be interesting to explain observations.

Very good point, thank you. We analyzed this in great detail and found viral RNA copies in the brain of LAV infected K18 mice only in the two animals with the highest clinical score (Fig 1C and 1E). This shows that the overexpression of ACE2 in the K18 model is a problem even for attenuated viruses. In general, in K18 and chACE2 mice and found a strong correlation between viral genome copies and interferon beta RNA expression (as marker of pathology/ inflammation) in the brain, as well as with the clinical score of the mice upon infection. **(New Sup Fig 1)** We adjusted the main text accordingly in **line 203-205.**

ll.214: The authors mention a perspective of intranasal vaccination of LAV. Where IgA levels checked as well? Antibodies are mentioned in the methods section, but mucosal immunity is not in the results. Would be very interesting to look at that aspect.

We checked for IgA in the BAL of the mice as well by FACS and ELISA. However, we only found very low IgA level upon infection with both viruses in the current settings. In contrast to IgG, the induction of IgA relies on local antigen expression and the recruitment of local IgA-producing plasma cells. Due to the attenuated nature of the LAV as well as the potent control of wt and LAV infection in young mice, this local antigen levels might not be high

enough to detect substantial amounts of IgA in the large volumes of our BAL samples. In follow up studies, we will ask whether increasing amounts of inoculate might lead to a stronger IgA response and will then include the complete IgA data in the paper.

ll.245: The authors state that viral genome only slightly differs between old and young inoculated mice. However, disease severity seems very different. Also here, further analysis would benefit the overall message and conclusions of the paper.

We thank the reviewer for bringing up this very interesting point. The age-dependent enhanced SARS-CoV2- pathology has been observed previously, in mice and men. However, the exact mechanisms causing the aggravated pathology in the elder is not understood. In mice, Beer and colleagues (PMID: 36129445), for example, found that the age-dependent severity was “associated with (i) lack of an early and well-coordinated innate and adaptive immune response, (ii) markedly increased viral load, and (iii) late inflammatory response”. While we also find higher viral loads in aged mice compared to young mice **(new Fig. 6D)**, we only observe minor differences in ISG induction upon infection between old and young mice (Fig. 4; Fig. 7). Thanks to the current study, we will be able to use the chACE2 model in the future to contribute to a better understanding of age-dependent pathology. We updated the text accordingly (**line 377-399).**

Fig.3D: There is still a lot of viral genome (maybe no infectious virus?) in the lungs of vaccinated animals. How can you be sure, if no data of histopathology are available, that your vaccine is really safe?

Or data on both infectious virus titer and genome copies show a fast decline of LAV over the course of 7 days, with very mild or no pathology in infected animals, even in highly susceptible aged animals. After 25 days, LAV RNA levels were on low background numbers in these animals (Fig. 9). Considering a 100- to 1000-fold drop in infectious virus titer compared to RNA genome levels **(new Fig. 6C, 6D),** we think it is highly unlikely that LAV replication can become active again, especially upon induction of anti-SARS-CoV-2 immune response as describe here. In addition, we added histopathology data for aged mice upon infection with LAV and WT (**new Fig. 8)**

ll.268: Can the authors explain why Delta was chosen as heterologous challenge? This variant is not of relevance anymore and the authors mention immune-escape of Omicron variants and

therefore limitations of spike mRNA vaccines in the intro. Wouldn't it have been a better proof of concept to use an Omicron variant as heterologous challenge to really prove an added value of your vaccine?

To test whether LAV Nsp16 protects against heterologous replication, we chose the Delta strain since it was readily available and we just aimed to proof the concept of blocking a heterologous infection. Of course, it would have been nicer to have the latest Omicron variant included in the analysis. However, since new SARS-CoV2 (sub)-variants are constantly emerging, we would have been always late to the game and therefor decided to settle for the well-characterized Delta variant to proof the point.

Biggest issue of the manuscript is that data of young vs old chACE2 mice are barely comparable. However, this distinct phenotype remains one of the main observations of the manuscript. One issue that young mice were sacrificed on day 2,4 and 7 (Fig 3). However, old mice were then chosen to be sacrificed on day 5. Figure 6 compares young vs old, yet looking at 2 different dpi (4 vs 5). In addition, 2 animals reached humane endpoints even earlier on (2 and 3 dpi). Despite of n=4, only 3 datapoints are shown for aged animals. Meaning that we look at data from 3 different timepoints of a single animal comparing to a group of 4 from yet another timepoint. This makes comparison and conclusions impossible, which is relevant for figure 6, 7 and 8. In your figure 3D you indicate the relevance of viral kinetics and how significant changes are over just 2 days. Therefore, none of the conclusion drawn from these data are valid due to n=1.

The reviewer is right. To better compare the data from young and old chACE2 mice, we repeated the infection experiments with additional groups of aged chACE2 mice. Animal numbers and time points of infection are comparable to the experiments with young mice. **(New Fig 6)**

Minor

Reviewer #1

-Overuse of general statements. Phrases like "Strikingly…" and "Together, we show…" are more appropriate for the discussion; the abstract should remain objective and concise.

The reviewer is correct, we adjusted the wording in the abstract.

- Clarify claims such as "problems of vaccine strategies targeting solely the S protein became visible" is vague. Specify what problems (immune escape, reduced efficacy, …). "huge number of sub lineages" is informal. Consider "extensive diversification of Omicron sublineages".

Thanks for spotting this. **We addressed the passage in the introduction**

- The introduction refers on age-related immune decline but could benefit from a mechanistic discussion such as immunosenescence or impaired IFN signaling (1-2 sentences).

We added the information in the introduction. **Line 106-116**

- What advantages does the chimera offer vs full human ACE2? Is this discussed in the Discussion section? Not clear in the introduction as "model justification".

We first introduce the chACE2 mouse model in **lines 175 ff** and explain its advantage.

- The statement in lines 131-132 is not entirely accurate, as wild-type mice have been shown to be susceptible to infection with certain SARS-CoV-2 variants, including B.1.351/Beta and BA.1.1/Omicron, as reported by Tarrés-Freixas et al. (Front Microbiol. 2022 May 4;13:840757. doi: 10.3389/fmicb.2022.840757. eCollection 2022).

The reviewer is correct, we updated the text and included the citations in **line 141-145**

- Line 137: please replace "SARS-CoV-1" with "SARS-CoV", as the correct nomenclature for the virus identified in 2002-2003 is "SARS-CoV"

We changed it to SARS-CoV.

- Line 141: please replace "qRT-PCR" with the correct terminology "RT-qPCR", which is the standard abbreviation for revers transcription quantitative PCR.

We changed it to RT-qPCR.

*- Line 142-143: the statement regarding enhanced tissue damaged observed in the BAL is misleading, as BAL is a fluid sample. While the figure legend notes that protein concentration in BAL was used as a surrogate marker for tissue injury, this rationale should also be explicitly stated in the results section. Additionally, it would be important to clarify why histopa*thological analysis of lung tissue was not performed to directly evaluate tissue damage.

We rewrote and clarified the section in **line 145-147, 315-320**

- Only viral RNA quantification has been performed; was infectious virus titration (e.g., plaque assay or TCID50) conducted to confirm the presence of replication-competent virus?

We quantified viral genomes most of the time since it is more compatible with harvesting for our other analysis (ISG PCR etc) and therefore saves animals. However, we also validated the RNA genome analysis by comparing it with life virus titer. We added the analysis in **new Figure 6C and 6D.**

- Figure 1E: is there any data available on the progression of disease in this animal model beyond 7 dpi with LAVNsp16? Specifically, could the onset or severity of disease be delayed?

We analyzed mice up to four weeks postinfection and didn't find any long-term effects. This shows for example in **Fig. 9,** where LAV immunized but not infected animals were observed for 4 weeks in total.

- Although the authors demonstrate that chACE2 mice support replication of both wt SARS-CoV-2 and the attenuated strain (Line 179), with clear differences in pathogenicity (absence of neuroinvasion and brain viral RNA), the authors rely on viral RNA quantification. It is not clear whether infectious virus titration was performed which is critical to confirm active replication and/or attenuation.

Please see above. We added the analysis in **new Fig. 6C, 6D.** Viral RNA titers nicely correlate infectious virus titer.

- Figure 3E interpretation: the absence of viral RNA in the brain is crucial, but histopathological confirmation would be valuable to rule out subclinical neuroinflammation.

The reviewer made a good point. To address subclinical neuroinflammation in the brain of infected mice, we analyzed ISG induction using highly sensitive RT-qPCR**. New Sup Fig 1.**

- The authors provide flow cytometry data showing innate immune cell infiltration and RT-qPCR data supporting local immune activation, but this reviewer questions the rationale for using RT-qPCR to assess IFNbeta and ISGs expression instead of flow cytometry to measure protein, which would provide more direct evidence of cytokine production and cellular activation.

We do agree with the reviewer that analyzing the cytokine production is a good way of analyzing innate immune activation. However, we also think that analyzing ISG transcript

upregulation as a marker of innate immune activation is well established in the field and, in combination with analyzing cellular and humoral immune responses, is well suited to analyze early innate responses upon infection in these mice

- The authors report anti-S IgG levels (Line 211-234) but do not assess neutralizing capacity, which is the gold standard for measuring neutralizing antibodies, thus, the ability to block virus infection.

Very valid point. We included a neutralization assay in the revised manuscript.

New Fig. 5F.

- Also, for aged mice (Lines 235-263) histological analysis of lung will support the claim of reduced tissue damage in those infected with LAVNsp16.

The author is right and we included the data in **new Fig 8.**

- The reduced ISG induction and immune cell infiltration are consistent with attenuation. However, age-related differences in baseline immune status should be discussed.

Good point raised by the reviewer. For ISGs, for example, our analysis shows the fold-increase after infection, therefore, baseline immune status doesn't show and the results are independent of possible differences in baseline immunity in aged animals. Thus, we reviewed our ISG expression data retrospectively and could not detect any endogenous upregulation of ISGs in aged naïve animals compared to young animals. A closer inspection of the role of immune -aging in those animals will be very interesting in the future.

- Again, in the section "homologous or heterologous SARS-CoV-2 challenge" (Lines 265-284): viral replication in BAL and lungs was assessed via RT-qPCR and no infectious titers are provided. Quantifying replication-competent virus is essential to validate vaccine efficacy.

We also validated our analysis by comparing viral RNA level with life virus titer. We added the analysis in **new Fig 6C, 6D** and found that viral titers in general nicely corresponded to infectious titer.

- The Delta variant challenge is described as resulting in no disease within four days, suggesting slower replication, but this interpretation requires caution. Longer periods post-challenge (7-10dpi) would be necessary to fully assess pathogenicity and vaccine protection.

Indeed, technically we cannot completely exclude a really slow Delta virus replication despite immunization (and becoming pathogenic only after 7-10 dpi). However, we think that this is highly unlikely. First and foremost, Delta RNA content in BAL and lung of these mice look exactly like that in mice after homologous infection. For both viruses, we do see a drastic drop in viral RNA level upon immunization suggesting that the immunization is working and effectively blocking heterologous RNA replication. Secondly, the complete loss of viral RNA (levels are at background level of mock infected animals) of Delta and Wuhan RNA makes it in our eyes highly unlikely that viral replication "bounces back" to high levels in these animals after another 3-5 days. Thirdly, experiments by Ye and colleagues using a different animal model but a similar LAV approach (SARS-CoV-2 lacking Nsp16) confirm our findings that such an LAV can protect against heterologous challenge (Ye et al., Cell Mol Immunity (11)).

Reviewer #2

1. Line 216. "Corelate" should be "correlate"

Changed in **line 226**

2. Fig. 6C and 6D are not referenced in the text.

Added references in **line 251**

3. Growth of SARS-CoV-2 wt and LAV in nasal turbinates is not discussed.

Of course, deeper analyses of the upper respiratory tract would have been informative, but technically not easily done in small animal models, such as mice. Furthermore, due to the inoculum dosage of 30 µl under ketamin/xylazine anesthesia, the main target tissue is the lower respiratory tract, which was extensively monitored here. However, we agree with the reviewer and will look at the upper the respiratory tract such as nasal turbinates. In future studies a lower volume in combination with isoflurane inhalation might be worth to consider, but needs a new animal license, which is out of scope for this study.

4. Fig. 6H. For clarity and consistency, "huACE2" should be "chACE2" and "B6" should be "mACE2".

We thank the reviewer for spotting the lack of clarity and rephrased in western blot figures accordingly **(new Fig. 2D, 6H)**

5. Fig. 8E and 8F. Lines 280-281. To conclude suppression to background levels in LAV-immunized and non-challenge mice, samples from mock-immunized and non-challenge controls should be included.

PCR on non-immunized, non-challenge samples did not result in any amplification product. Thus, we did not include it in Figures 8e and 8F, which display copy numbers. In addition, we feel that immunized non-challenge samples is the better control because it displays the true background of the analysis "naïve vs immunized". **We rephrased this section to emphasize the background in non-challenge samples (line 290).**

6. Line 297. "live attenuate" should be "live attenuated"

Changed in **line 297**

Reviewer #3

Title: I would recommend to write out chACE2

We changed the title

Ll.44: Throughout the manuscript authors use the term "long-term consequences" and refer to an immunological response upon vaccination. To me the use of this term is misleading since it implies rather long-term effects as observed for Long COVID. I would recommend re-wording.

We changed the wording to exclude misinterpretations.

ll. 289: The authors mention that other chimericACE2 models were established previously (Ref29-32). It would be good to state the novelty of their own model here. What is the added value/difference/unique aspect, compared to others?

We emphasized the unique features of our **model in line 319-332**

ll.633 vs 660: If K18hACE2 mice should be compared with the chACE2 mice, why were different inoculation doses used?

Due to artificial overexpression of hu ACE2 in K18 mice, only low levels of inoculum are needed for high viral titers and severe pathogenicity. In the context of natural receptor expression, however, lower viral titers and no pathogenicity were observed when

inoculated with the same amount of virus (not shown). To make sure, we are not missing out on pathogenicity in chACE2 mice, we even increased the inoculum by 70-fold (resulting in similar high viral titers, but still no pathogenicity), We adjusted the wording in **lines 190-192.**

Throughout the whole manuscript the authors refer to viral titers. Please change to viral genome (titers), since readers can otherwise confuse it with life virus data.

We thank the reviewer for spotting the lack of clarity and changed the wording accordingly. On top, and as suggested by the reviewers, we also determined the viral titers to corroborate our findings and show that life viral titers and viral copy numbers nicely correlate **(new Fig 6C, 6D)**

ll. 639: A protein measurement of BAL is used as surrogate for tissue damage. Is that method been published before and are there actual proven correlations with damage?

That method is frequently used to assess epithelial barrier and tissue damage and has been published before, for example in PMID: **21094033 or PMID: 40017475.** We added the references in the text.

ll.668: Panel E is not referenced in this caption.

Thank you very much. We added the reference for (E).

Fig.8: It is indeed surprising to see (as authors also mention in the discussion) that almost no changes are observed during an infection with Delta. Also here, pathology and further downstream analysis could support explanations for this observation.

We agree with the reviewer, it is very interesting and we will include detailed analysis on Delta and other subtypes in a follow-up study.

Figures of the supplement were not referenced to in the main text.

Thank you very much for spotting this. We added references to Sup Figures in the text.
